# Structural neuroimaging differentiates between depressed bipolar disorder and major depressive disorder patients: a machine learning study

**DOI:** 10.1192/j.eurpsy.2023.1280

**Published:** 2023-07-19

**Authors:** F. Calesella, F. Colombo, B. Bravi, L. Fortaner-Uyà, C. Monopoli, E. Tassi, E. Maggioni, I. Bollettini, S. Poletti, F. Benedetti, B. Vai

**Affiliations:** 1 Vita-Salute San Raffaele University; 2 Psychiatry and Clinical Psychobiology Unit, Division of Neuroscience, IRCCS San Raffaele Hospital; 3 Department of Neurosciences and Mental Health, IRCCS Fondazione Ca’ Granda Ospedale Maggiore Policlinico, Milano, Italy

## Abstract

**Introduction:**

Depression is the predominant mood alteration in bipolar disorder (BD), leading to overlapping symptomatology with major depressive disorder (MDD). Consequently, in clinical assessment, almost 60% of BD patients are misdiagnosed as affected by MDD. This calls for the creation of a framework for the differentiation of BD and MDD patients based on reliable biomarkers. Since machine learning (ML) enables to make predictions at the single-subject level, it appears to be particularly suitable for this task.

**Objectives:**

We implemented a ML pipeline for the differentiation between depressed BD and MDD patients based on structural neuroimaging features.

**Methods:**

Diffusion tensor imaging (DTI) and T1-weighted magnetic resonance imaging (MRI) data were acquired for 282 depressed BD (n=180) and MDD (n=102) patients. Axial (AD), radial (RD), mean (MD) diffusivity, and fractional anisotropy (FA) maps were extracted from DTI images, and voxel-based morphometry (VBM) measures were obtained from T1-weighted images. Each feature was entered separately into a 5-fold nested cross-validated ML pipeline differentiating between BD and MDD patients, comprising: confound regression for nuisance variables removal (i.e., age and sex), feature standardization, principal component analysis, and an elastic-net penalized regression. The models underwent 5000 random permutations as a test for significance, and the McNemar’s test was used to assess whether there was any significant difference between the models (significance threshold was set to p<0.05).

**Results:**

The performance of the models and the results of the permutation tests are summarized in Table 1. McNemar’s test showed that the AD-, RD-, MD-, and FA-based models did not differ between each other and were significantly different from the VBM.

**Table 1.** Models’ performance and p-value at 5000 permutation test.

**Feature**	**Overall accuracy**	**MDD specifictiy**	**BD sensitivity**	**p-value**
VBM	0.61	0.38	0.74	0.058
AD	0.78	0.65	0.86	<0.001
FA	0.79	0.61	0.89	<0.001
MD	0.79	0.63	0.88	<0.001
RD	0.79	0.63	0.88	<0.001

**Conclusions:**

In conclusion, our models differentiated between BD and MDD patients at the single-subject level with good accuracy using structural MRI data. Notably, the models based on white matter integrity measures relying on true information, rather than chance.

**Disclosure of Interest:**

None Declared

